# Unveiling the metric structure of internal representations of space

**DOI:** 10.3389/fncir.2013.00081

**Published:** 2013-04-26

**Authors:** Federico Stella, Erika Cerasti, Alessandro Treves

**Affiliations:** ^1^Neuroscience, SISSATrieste, Italy; ^2^Department of Human Physiology and Pharmacology, Università “La Sapienza”Rome, Italy

**Keywords:** spatial representations, place cells, CA3, decoding, neural network, information theory, metric space

## Abstract

How are neuronal representations of space organized in the hippocampus? The self-organization of such representations, thought to be driven in the CA3 network by the strong randomizing input from the Dentate Gyrus, appears to run against preserving the topology and even less the exact metric of physical space. We present a way to assess this issue quantitatively, and find that in a simple neural network model of CA3, the average topology is largely preserved, but the local metric is loose, retaining e.g., 10% of the optimal spatial resolution.

## Introduction

CA3 neurons in rodents develop their selectivity for certain portions of any new environment (O'Keefe and Dostrovsky, 1971; Wilson and McNaughton, 1993) through an ongoing unsupervised learning process, probably driven by the dentate gyrus (Treves and Rolls, 1992; Leutgeb et al., 2007) which is random in nature and which generates a non-trivial mapping between the external input (the position of the animal in that environment) and the response of individual cells. The position, the shape and the number of the fields developed by each CA3 cell are thus not captured by any simple rule of organization (Park et al., 2011). This is true also for non-spatial stimuli, which are most probably deposited in the hippocampal memory store in a similar fashion (Komorowski et al., 2009; Naya and Suzuki, 2011; Tort et al., 2011). Also in this case, we expect neurons to develop random profiles of activation to stimulus features, spanning random regions of feature space (Quiroga et al., 2005).

What is specific about physical space, and makes it different from other correlates of neuronal activity, is its intrinsic topographical structure. A set of spatial stimuli are naturally endowed with a canonical topology and with a continuous metric, defined by the relative position of locations in the environment. These stimuli thus span a multi-dimensional manifold, which, in the typical experimental situation of a recording box, is two-dimensional and Euclidean. How “spatial” is the internal representation generated in CA3? How much of the external metric is preserved inside the brain? (Samsonovich and McNaughton, 1997; Stringer et al., 2002; McNaughton et al., 2006).

One may pretend to ignore the real-world metric, and study the metric of the virtual manifold established by the patterns of neuronal activity with which physical space has been associated (Muller and Stead, 1996; Muller et al., 1996). How? In CA3 the movements of an animal traversing an environment elicit, on repeated trials, a distribution of responses which one can use to define distances between pairs of locations (Brown et al., 1998; Deneve et al., 1999; Averbeck et al., 2006), in terms e.g., of the mean overlaps in the corresponding distributions of population vectors, and one can analyze the overall structure of such pair-wise distances in geometric terms.

Ideally a faithful mapping of space should produce isometric representations, i.e., whose relationships mirror the relationships induced in real space by the Euclidean metric (Curto and Itskov, 2008). Such an ideal mapping is, however, unfeasible with any finite neuronal population, even more so with a random self-organization process (Tsodyks and Sejnowski, 1995; Hamaguchi et al., 2006; Papp et al., 2007; Roudi and Treves, 2008). But how to assess the degree of deviation from isometricity?

Spatial representations in CA3 depend, of course, not just on the physical structure of external space but also on how it is *perceived* by the animal, and on the effective dimensionality of the representation, as spanned by animal behavior (Hayman et al., 2011; Ulanovsky and Moss, 2011). Distant locations might be seen as similar or confused altogether, irrelevant dimensions might be ignored, e.g., on a linear track, the relative distance between locations might be distorted, not all the locations might be assigned a representation in the population, etc. In a word, the Euclidean nature of external space may be altered arbitrarily. To start with, it is useful, however, to remove such arbitrariness and consider a model situation in which there is nothing but the Euclidean metric of physical space to be represented, through self-organization. This is what we set out to do in this study. While in a companion paper (Cerasti and Treves, under review) we focus on characterizing the local smoothness of these representations, and how it scales with the size of the network, here we aim to quantify their metric content.

In Treves (1997), and later papers, e.g., Treves et al. (1998), Ciaramelli et al. (2006), and Lauro-Grotto et al. (2007), a *metric content* index was introduced in order to characterize the amount of perceived metric in the representation of a discrete set of stimuli, such as faces. It was shown empirically that such metric content index is almost invariant as one varies the sample of cells used to assess the representation. Thus, it approaches the role of an objective or intrinsic measure, insensitive to the procedure used to extract it (e.g., how many and which cells are recorded in a particular experiment). Can a similar descriptor be applied to the representation of real space?

Although physical space is low dimensional, each spatial variable can span during everyday behavior a small or large interval along any of the dimensions and, crucially, can do so continuously. To actually define a set of distinct locations, for data analysis, one has in practice to discretize space in a finite number of bins and to assign to each of them a reference population vector, resulting from averaging over the activity expressed when in that bin (the actual procedure to perform this average can vary). One would want these bins to be as small as possible, to retain part of the continuity compromised by the binning. On the other hand, any refinement of the bin resolution leads unavoidably to a low sampling problem.

This “curse of dimensionality” (Golomb et al., 1997; Panzeri et al., 2007) (where the dimensionality referred to is not of space itself, but of multiple locations in space) limits the current feasibility of the metric content analysis of a set of real, experimental data. The length of a recording session necessary to properly sample the distribution of population vectors tends to be prohibitive. One may, however, turn to computer simulations, which can be as long as needed, to produce the data necessary for the analysis.

## Materials and methods

### Basic model

The model we consider is, as in Cerasti and Treves (under review) an extended version of the one used in our previous study (Cerasti and Treves, 2010), where the firing rate of a CA3 pyramidal cell, η_*i*_, was determined, as the one informative component, by the firing rates {β} of DG granule cells, which feed into it through mossy fiber (MF) connections. The model used for the neuron was a simple threshold-linear unit (Treves, 1990), so that the firing of the unit results from an activating current (which includes several non-informative components) and is compared to a threshold:

(1) $$\eta_{i}(\vec{x})=g{\left[\sum_{j}c_{ij}^{\text{MF}}J_{ij}^{\text{MF}}\beta_{j}(\vec{x})+{\tilde{\delta }}_{i}-\tilde{T}\right]}^{+}$$

where *g* is a gain factor, while [·]^+^ equals the sum inside the brackets if positive in value, and zero if negative. The effect of the current threshold for activating a cell, along with the effect of inhibition, and other non-informative components, are summarized into a single subtractive term, with a mean value across CA3 cells expressed as $\tilde{T}$, and a deviation from the mean for each particular cell *i* as ${\tilde{\delta }}_{i}$, which acts as a sort of noise; threshold and inhibition, in fact, while influencing the mean activity of the network, are supposed to have a minor influence on the coding properties of the system. In the earlier reduced model, however, $\tilde{T}$ and ${\tilde{\delta }}_{i}$ also included the effect of other cells in CA3, through RC connections, and that of the perforant path, both regarded as unspecific inputs—this based on the assumption that information is driven into a new CA3 representation solely by MF inputs.

In Cerasti and Treves (under review) and in this study, instead, since we are interested in the ability of the RC system (Amaral et al., 1990; van Strien et al., 2009) to express spatial representations, we separate out the RC contribution, and redefine $\tilde{T}$ and ${\tilde{\delta }}_{i}$ into *T* and δ_*i*_—which sum the remaining unspecific inputs, including the perforant path, not analyzed here:

(2) $$\eta_{i}(\vec{x})=g{\left[\sum_{j}c_{ij}^{\text{MF}}J_{ij}^{\text{MF}}\beta_{j}(\vec{x})+\sum_{k}c_{ik}^{\text{RC}}J_{ik}^{\text{RC}}\eta_{k}(\vec{x})+\delta_{i}-T\right]}^{+}$$

Connections between cells are indicated by the fixed binary matrices {*c*^*MF*^}, {*c*^*RC*^}, whose non-zero elements (which take value 1) represent the existence of anatomical synapses between two cells. The synaptic efficacies are instead indicated by the matrices of weights {*J*^*MF*^}, {*J*^*RC*^}, whose elements are allowed to take positive values. The notation is chosen to minimize differences with our previous analysis of other components of the hippocampal system (e.g., Treves, 1990; Kropff and Treves, 2008).

The perforant path inputs from Entorhinal Cortex are not explicitly included in the model, in line with the hypothesis that they relay the cue that initiates the retrieval of a previously stored representation, and have no role in the storage of a new representation and in defining the properties of the attractors in CA3. This perspective has been theoretically described in (Treves and Rolls) and has found experimental support (Lassalle et al., 2000; Lee and Kesner, 2004).

The firing rates of the various populations are all assumed to depend on the spatial position $\vec{x}$ of the animal; and the time scale considered for evaluating the firing rate is of order the theta period, about 100 ms, so the finer temporal dynamics over shorter time scales is neglected. To be precise, in the simulations, we take a time step to correspond to 125 ms of real time, or a theta period, during which the simulated rat moves 2.5 cm, thus at a speed of 20 cm/s. This is taken to be an average over a virtual exploratory session, familiarizing with a new environment.

### The storage of new representations

The important novel ingredient that was introduced by (Cerasti and Treves, 2010), and that makes the difference from previous models of self-organizing recurrent networks, is a realistic description of the patterns of firing in the inputs, i.e., in the dentate gyrus. As the virtual rat explores the new environment, the activity $\beta_{j}(\vec{x})$ of DG unit *j* is determined by the position $\vec{x}$ of the animal, according to the expression:

(3) $$\beta_{j}(\vec{x})=\sum_{k=0}^{Q_{j}}\beta_{0}e^{-{\left(\vec{x}-{\vec{x}}_{jk}\right)}^{2}/2\sigma_{f}^{2}}$$

The firing rate of the granule cells is then a combination of *Q*_*j*_ Gaussian functions, resulting in “bumps,” or fields in the firing map of the environment, centered at random points ${\vec{x}}_{jk}$. The environment is taken to have size *A*, and the fields are defined as all having the same effective size π(σ_*f*_)^2^ and height β_0_. *Q*_*j*_, which indicates the multiplicity of fields of DG cell *j*, is drawn from a Poisson distribution:

(4) $$P(Q_{j})=\frac{q^{Q_{j}}}{Q_{j}!}e^{-q}$$

with mean value *q*, which roughly fits the data reported by Leutgeb et al. According to the same experimental data, we assume that only a randomly selected fraction *p*_*DG*_ << 1 (here set at *p*_*DG*_ = 0.033) of the granule cells are active in a given environment. Hence population activity is sparse, but the firing map of individual active granule units need not be sparse [it would only be sparse if *q*π(σ_*f*_)^2^/*A* << 1, which we do not assume to be always the case].

The activity of DG units determines the probability distribution for the firing rate of any given CA3 pyramidal unit, once the connectivity level between the two layer has been fixed: {*C*^MF^_*ij*_} = 0, 1 with $P\left(C_{ij}^{\text{MF}}=1\right)=\frac{C^{\text{MF}}}{N_{DG}}\equiv c^{\text{MF}}$. In agreement with experimental data, we set *C*^*MF*^ = 50, a value in the range of the ones providing an optimal information transmission from DG to CA3 (Cerasti and Treves, 2010). The MF synaptic weights are set to be uniform in value, *J*^MF^_*ij*_ ≡ *J*, and similarly *J*^RC^_*ij*_ ≡ *J*^RC^_0_ initially. Subsequently, during the learning phase, RC weights are modified according to the simulated learning process and under the influence of the input coming from the MF connections. Following the simplified hypothesis that the MFs carry all the information to be stored without contributing anything to the retrieval process, which is left to the recurrent collateral, MF weights are kept fixed to their initial values *J*; note that we have found, in our earlier study that MF connections appear to be inadequate, even when associatively plastic, to support retrieval of spatial representation (Cerasti and Treves, 2010).

The connectivity among CA3 cells is given by the matrix {*C*^RC^_*ij*_} = 0, 1 with $P\left(C_{ij}^{\text{RC}}=1\right)=\frac{C^{\text{RC}}}{N_{CA3}}\equiv c^{\text{RC}}$, where *C*^*RC*^ = 900 in most simulations. The activity of the network is regulated by the constraint we impose on its mean and on its sparsity *a*_*CA*3_, i.e., the fraction of the CA3 units firing significantly at each position, which is an important parameter affecting memory retrieval [(Treves, 1990); more precisely, *a*_CA3_ = 〈η_*i*_〉^2^/〈η^2^_*i*_〉]. Here we set the sparsity of each representations as *a*_*CA*3_ = 0.1, in broad agreement with experimental data (Papp et al., 2007), and at each time step we regulate the threshold *T* accordingly, to fulfill such requirement, while keeping the mean activity 〈η_*i*_〉 = 0.1 by adjusting the gain *g*.

### Recurrent collateral plasticity

During the learning phase, the activity of CA3 is driven by DG inputs, and RC connections contribute through weights uniformly set to their initial value *J*^RC^_0_. While the virtual rat explores the environment, RC weights are allowed to change according to an associative “Hebbian” learning rule, such that the total change in the synaptic weights is given as a sum of independent terms

(5) $$\Delta J_{ij}^{\text{RC}}(t)=\gamma \eta_{i}(t)\left(\eta_{j}(t)-\Lambda_{j}(t)\right)$$

where Δ*J*_*ij*_(*t*) indicates the variation of the connection weight between cells *i* and *j* occurring at a given time step *t*, η_*i*_, and η_*j*_ are the postsynaptic and presynaptic firing rate, while γ is the learning rate. This associative learning rule includes the contribution of a trace, Λ, of the recent past activity of the presynaptic cell, defined as

(6) $$\Lambda_{j}(t)=\frac{1}{\tau }\sum_{t_{s}=1}^{\tau }\eta_{j}(t-t_{s})$$

where τ is taken equal to 14 time steps (1750 ms). RC weights are forced to be non-negative, so they are reset to zero each time they become negative. Moreover, the total of the synaptic weights afferent to a single postsynaptic CA3 cell is normalized at the end of the learning process, so that ∑^*C*^RC^^_*j* = 1_ *J*^RC^_*ij*_ = 1 per each CA3 cell. In words, the synaptic plasticity on recurrent connections allows the system to store the information about the current environment conveyed by MF inputs; such information is expressed in the form of place-like patterns of activity in CA3 units, and the Hebb-like learning rule strengthens the connections between units that show overlapping fields.

### Threshold setting in CA3

CA3 units fire according to Equation (2), with the threshold *T* hypothesized to serve to adjust the sparsity *a*_*CA*3_ of CA3 activity to its required value. The sparsity is defined as

(7) $$a_{\text{CA3}}={\left(\sum_{i}\eta_{i}(\vec{x})\right)}^{2}/\sum_{i}\eta_{i}^{2}(\vec{x})$$

and it is set to *a*_*CA*3_ = 0.1. This implies that the activity of the CA3 cell population is under tight inhibitory control.

### Simulations

The mathematical model described above was simulated with a network of 45,000 DG units and 1500 CA3 units. A virtual rat explores a continuous two dimensional space, intended to represent a 1 sqm square environment but realized as a torus, with periodic boundary conditions. In each time step (of 125 ms) the virtual rat moves 2.5 cm in a direction similar to the direction of the previous time step, with a small amount of noise.

Before and during the learning session, all recurrent connections weights take the same value *J*^RC^_0_; after the learning phase, they take the values resulting from the sum of all modifications occurred during the session, and described by Equation (5), with learning rate γ. The trajectory of the virtual rat during the learning session is a random path, extended over a time long enough for it to effectively visit repeatedly all possible locations in space: 10,000 time steps to cover the entire environment. This is taken to correspond to about 20 min of exploration in real time. Such synaptic modifications start to have an effect on the CA3 firing rate only at the end of the learning session, when the RC weights are updated to their new values.

After the learning phase for the CA3 network, we run a training phase for our decoding algorithm, during which we build the so called activity templates (Rolls and Treves, 2011): the environment is discretized in a grid of 8 × 8 locations and to each of these bins we associate a reference population vector. This reference vector for a bin is obtained averaging the activity of the network in all the time steps that the rat spends in that bin during a simulation of 100,000 time steps.

Subsequently a test phase is run. This time the rat is left wandering on a random trajectory through the environment and at each time step the activity generated in the network is compared to the templates previously defined. The dynamics is analyzed when the input coming from the DG units is either on, to characterize externally driven representation, or turned off, to characterize instead memory driven attractors (Wills et al., 2005; Colgin et al., 2010). The noise level we use is kept very low (δ = 0.002), as we are more interested to probe the microstructure of the spatial representation rather than to test its robustness. To have a roughly even coverage of the surface of the environment and to produce a sufficient number of visits to each one of the locations in which it is divided, simulations are run for 400,000 time steps (nearly 35 h of virtual rat time).

For simulations without DG direct input, aimed at describing attractor properties, in each step of the virtual rat trajectory, activity is allowed to reverberate for 15 time steps; with a full DG input during the first one, an input reduced to 1/3 during the second, and to 0 for the remaining 13 time steps. The final configuration attained by the system after this interval is then observed and the rat is moved to the next position and the procedure is repeated.

## Analysis and results

### Global metric

What does the global structure of the CA3 representation generated in our simulations look like? One may start addressing this question by using the most comprehensive measure of activity in the network. In our simulations, templates are generated using the entirety of the cells and averaging their activity over the spatial extent of the relative bin. They are the fingerprints of the representation of the environment contained in CA3. Templates are vectors in a high-dimensional space, namely the number of dimensions corresponds to the number of units in the network, 1500 in the simulations we use for the analysis. To visualize the configuration of these vectors and their arrangement, one should use some procedure to reduce their dimensionality and to produce a readable picture. One can use the correlation between the vectors associated to different positions to construct a *similarity matrix* containing the relative distance of all the vector pairs. Multidimensional scaling (MDS) uses this similarity matrix to assign positions to the templates in a Euclidean space of a specified dimension (in our case, 3-dimensional), so as to best preserve the ordering of the distances in the distance matrix. One can then directly compare the configuration obtained from the algorithm to the topology of the external environment, which, in the case of our simulations that make use of periodic boundary conditions, is a two-dimensional torus.

**Figure 1:**
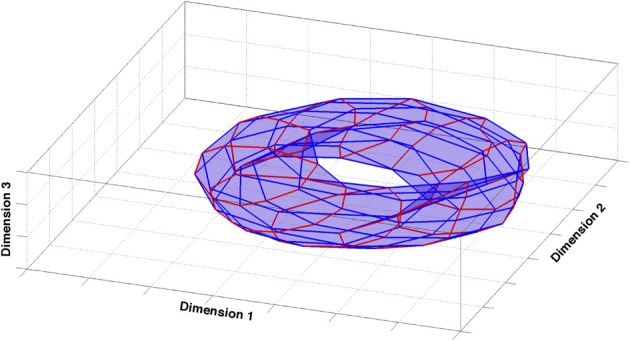
**Multidimensional scaling analysis.** The figure shows the 3-dimensional configuration of template vectors based on pairwise correlations obtained by multidimensional scaling [Sammon criterion (Sammon, 1969)], applied to the spatial representation produced by the model CA3 network. The configuration closely reproduces the original similarity matrix (Stress = 0.04). Red and blue lines correspond to orthogonal directions in the external environment.

In fact, also configurations produced by a metric MDS [Sammon algorithm (Sammon Jr), Stress = 0.04] tend to have a torus-like topology (Figure 1). The global properties of the model CA3 representation thus faithfully reproduce those of external space, at least in terms of average templates. This assessment, however, is only qualitative and it misses finer details that may hide at different levels of resolution. The actual responses of the network while the animal is traversing the environment are averaged away in building the templates. Moreover a measure based on the activity of the whole population does not provide indications as to the distribution of information sampled, in practice, from the few neurons which can be recorded simultaneously, leaving doubts as to whether such a global measure has any experimental relevance. We are then left with the problem of constructing a synthetic description of the representation that comprises information on its local properties, as gauged by observing a few units at a time.

### Decoding

To quantitatively assess the features of a neural representation of any set of stimuli we can rely on some standard procedures (Quian Quiroga and Panzeri, 2009). Decoding the spike trains emitted by a population of neurons, when one (*s*) of a set of stimuli is presented, means applying an algorithm that estimates, given the current spike train and those previously recorded in response to each stimulus, the likelihood for each (*s*') of the possible stimuli to be the current one. The stimulus for which the likelihood is maximal is the stimulus predicted on the basis of the chosen decoding algorithm. One repeats this all the times s is the current stimulus, to generate a table *P*(*s*' | *s*). The decoded stimulus is not necessarily the correct one, and a first measure of the nature of the representation is just the fraction of correct hits in the table, ∑_*s*_ *P*(*s* | *s*)*P*(*s*), where *P*(*s* | *s*) are just the diagonal elements of the confusion matrix. A more complex, yet more complete, measure is given by the mutual information I

(8) $$I=\sum_{r,s}P(r,s)\log_{2}\frac{P(r,s)}{P(r)P(s)}$$

which also reflects the distribution of errors, and thus provides further insight on the way the stimuli are encoded (Rolls and Treves). These two quantities, percent correct and mutual information, depend on the pool of neurons used to perform the decoding, and most crucially on the size of this pool. More cells obviously allow for better decoding.

The two quantities are not completely independent, as there are mutual constraints between them. At the same time, one does not completely define the other: given a certain percent correct there is a possible range of information values that depends on the way errors are distributed among incorrect locations. At one extreme, when there is no overlap between the representations of different stimuli, we expect errors to be distributed at chance: the “distance” between any pair of stimuli is maximal and effectively the same, and no non-trivial metric can be defined. Conversely, any non-uniform overlap influences the way errors are produced. The more the distribution of errors deviates from being flat, the more the representation contains overlapping and interfering elements.

### The spatial confusion matrix

During the test phase of our simulations, at each time step, the firing vector of a set of CA3 units is compared to all the templates recorded at each position in the 8 × 8 grid, for the same sample, in a test trial (these are the template vectors). The comparison is made by calculating the Euclidean distance between the current vector and each template, and the position of the closest template is taken to be the decoded position at that time step, for that sample. This procedure applies to our spatial analysis what has been termed maximum likelihood Euclidean distance decoding (where the distance between population vectors should not be confused with the distance between locations in the environment). The frequency of each pair of decoded and real positions are compiled in a so-called “confusion matrix.” Should decoding “work” perfectly, in the sense of always detecting the correct position in space of the virtual rat, the confusion matrix would be the identity matrix. The confusion matrix is thus *L*^2^ × *L*^2^ (64 × 64 for our simulations) and its dimension grows very fast when increasing the number of bins, requiring a prohibitively longer number of time steps to properly fill all its entries.

The confusion matrix gives us the value of the percent correct and of the mutual information. The size of the sample is then varied to describe the dependence of these quantities on the number of cells in the pool. We used samples of up to 256 units, a number which can be compared with the total population in our CA3 network, 1500 units.

### Metric content

For a given percent correct (*f*_cor_) there is in general, in a non-spatial paradigm, a certain range of possible amounts of information contained in the confusion matrix. Ideally the information should be comprised between a minimum value

(9) $$I_{\min}=\log_{2}S+f_{\text{cor}}\log_{2}f_{\text{cor}}+(1-f_{\text{cor}})\log_{2}(1-f_{\text{cor}})-(1-f_{\text{cor}})\log_{2}(S-1)$$

(where *S* is the number of elements in the stimulus set) corresponding to an even distribution of errors among all the possible stimuli, while the maximum is attained when all the errors are concentrated on a single incorrect stimulus

(10) $$I_{\max_{\text{bias}}}=\log_{2}S+f_{\text{cor}}\log_{2}f_{\text{cor}}+(1-f_{\text{cor}})\log_{2}(1-f_{\text{cor}})$$

However, this maximum corresponds to a systematic misclassification of the current location by the network. It might therefore be reasonable to assume that our system is an unbiased classifier, which implies that incorrect stimuli can at most be chosen as frequently as the correct one, and reformulate the previous maximum in the following terms

(11) $$I_{\max}=\log_{2}S+\log_{2}f_{\text{cor}}$$

The metric content index can then be defined, in such a non-spatial paradigm, as

(12) $$\lambda =\frac{I-I_{\min}}{I_{\max}-I_{\min}}$$

This is the measure considered in our previous studies. With this choice, though, we wouldneglect the intrinsic topological structure of spatial information. In fact, the high correlation existing between the representations of neighboring locations constrains the distribution of errors. We need to distinguish between errors originating from random correlations in the representation of distant, unrelated locations, and errors emerging form the continuity of the representation. The latter can be considered as structural to the representation and they are expected to be present around the correct location even in the case of optimal decoding, but with limited spatial resolution.

### Full and reduced matrix

**Figure 2:**
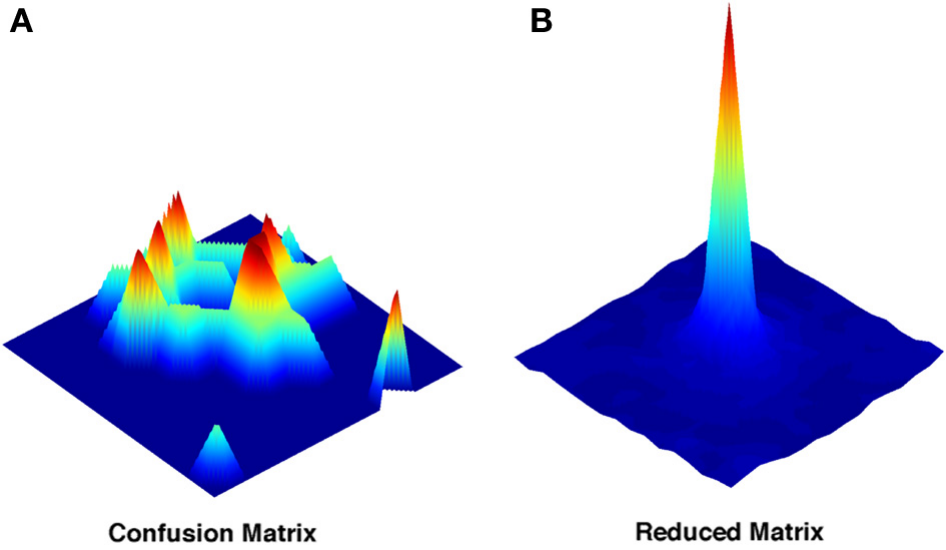
**Representative examples of the full and of the reduced confusion matrix. (A)** Decoding probability distribution for a single location extracted from the complete confusion matrix. **(B)** Decoding probability distribution obtained from the reduced matrix. Both are obtained with a sample size of *N* = 4. Color coding just reflects the relative heights of the points in the probability distributions.

The confusion matrix conveys information which is location-specific: we know for each of the locations how it was decoded during the test phase. The appearance of a typical example of these location-specific matrices shows how a decoding approach reveals characteristics of the representation otherwise overseen (Figure 2A). Far from being close to the real position, the decoded positions appear to be distributed in multiple locations over the environment, often far away from the correct spot. The pattern of distribution of decoding probability depends on the choice of the sampled neurons, and on the size of the sample, and it seems to lack any regular principle of organization.

If any regularity in the distribution of errors across positions exists, we can try to reveal it by extracting the translational invariant component of this distribution. By deriving a position-averaged version of the matrix (Figure 2B), we construct a simplified matrix *Q*_(*x* − *x*0)_, which averages over all decoding events with the same vector displacement between actual (*x*0) and decoded (*x*) positions. *Q*_(*x* − *x*0)_ is easily constructed on the torus we have used in all simulations, and it is only L × L, much smaller than the complete confusion matrix.

**Figure 3:**
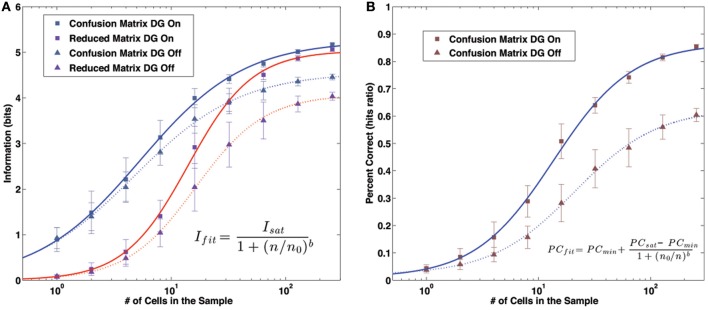
**Information and Percent Correct dependence on sample size. (A)** Information values. Markers: model data. Lines: fit curves. Gray markers/blue lines: information content of the confusion matrix. Purple markers/red lines: information content of the reduced matrix. Square markers/solid lines: results with the DG input active. Triangular markers/dashed lines: results without the DG input. **(B)** Percent Correct values. Markers: model data. Lines: fit curves, as in **(A)**.

The two procedures, given that the simplified matrix is obtained just by averaging the full confusion matrix after a row translation, might be expected to yield similar measures, but this is not the case (Cerasti and Treves, 2010). In fact the amount of information that can be extracted from the reduced matrix is significantly inferior to that of the full matrix, even if the difference decreases as the sample of neurons gets larger (Figure 3A). The discrepancy between the two measures reflects the presence of a distribution of errors which is not translational invariant. The distribution found in the reduced matrix instead represents the performance of the system when the effects of specific firing configurations are averaged away. Being quasi-randomly distributed, the errors of the complete matrix average out and produce a smooth, radially decreasing distribution around the central, correct position in the reduced matrix (Figure 2B). We are thus able to extract the average error dependence on the distance between two points, regardless of their specific position. The reduced matrix is the expression of the overlaps induced by the external metric of space together with the continuity of the internal place field representation.

All the quantities of interest, the information contained in the full and reduced matrix and the percent correct, have a dependence on the sample size which can be fit rather precisely by a sigmoid function. The function we use to fit the information is

(13) $$I_{\text{fit}}=\frac{I_{\text{sat}}}{1+{\left(n_{0}/n\right)}^{b}}$$

where *n* is the number of units in the decoding sample and *I*_sat_, *n*, and *b* are the fit parameters. An analogous form is used for the percent correct,

(14) $$f_{{\text{cor}}_{\text{fit}}}=f_{{\text{cor}}_{\min}}+\frac{f_{{\text{cor}}_{\max}}-f_{{\text{cor}}_{\min}}}{1+{\left(n_{0}/n\right)}^{b}}$$

The change in the convexity of the data is particularly evident when using a log scale for the number of unit in the sample (Figures 3A,B).

### The metric induced by an active DG

We can use the information contained in the reduced matrix to separate the effects of the external metric on the CA3 representation from the other sources of correlation. For this aim, the condition with the active DG is better suited, as we consider the original map imposed by the external input, before the modifications induced by the storage, which will be described in the following section.

**Figure 4:**
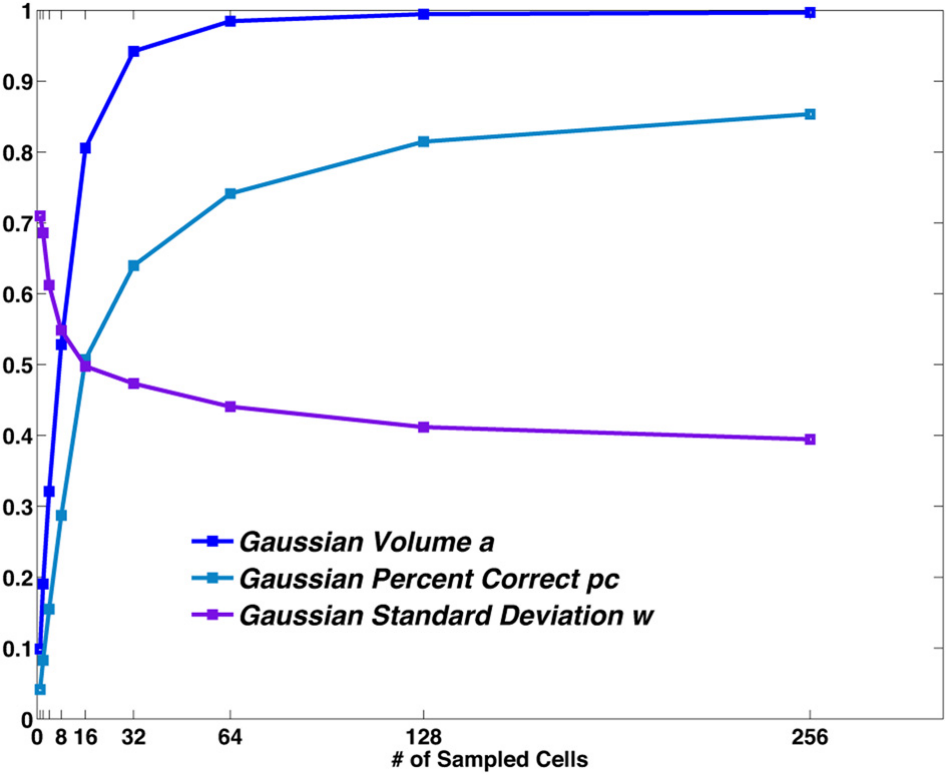
**The Gaussian distribution parameters vs. sample size.** The values obtained from the fit of the central bump of the reduced matrix, are plotted for different sample sizes. *Pc* and *a* are probabilities. *w* is measured in spatial bin units (each bin is 12.5 cm in size).

If we fit the distribution of values in the reduced matrix with a Gaussian we can extract the parameters describing the distance dependence of the errors, namely the height of the central peak (*pc*), the width of the distribution (*w*) and the total volume below the distribution (*a*). They correspond, respectively, to the number of correct hits, the spread of errors around the central location and to the proportion of decoding steps associated with a single location. These parameters depend on the sample size (Figure 4): a larger sample corresponds to a higher peak of the Gaussian (corresponding to an increase in the percent correct), to a lower standard deviation around the mean and to a higher total volume. It should be noted, however, that the observed width *w* bundles together the distribution of outright errors and the spread of correct but spatially imprecise responses.

A non-zero standard deviation σ, which can then be regarded as a component of the width *w*, expresses the difference between decoding a set of spatial stimuli and decoding a non-spatial one.

We can use this measure of the structural confusion between locations of the environment to reformulate our measure of metric content for the case of spatial information. We can argue that given a certain percent correct, the minimal information would be obtained when the decoding distribution corresponds to a Gaussian of width σ and total volume *a* centered on the correct location, plus the remaining (1 − *a*) evenly distributed on all the spatial bins. Analogously the maximal information attainable would correspond to the situation in which 1/*a* Gaussians of the same shape sit on the same number of different locations (one of them, of course, should be placed on the correct one). Indeed this is a first maximum, and it corresponds to the maximal unbiased information defined above for the non-spatial case.

For the spatial case, however, these minimum and maximum information values largely reflect simply the definition of the reduced and full confusion matrix. They tell us about the procedure used in the analysis more than about the representations being analyzed. We can, however, go further, as we can also extract the limit toward the discrete case for our model, attained when the spatial resolution is optimal, i.e., the spatial code is precise, spatially exact. By sending the standard deviation σ to zero, in the limit, and by replacing the Gaussian distribution with a single peak of height *a* located in the central spot, we retrieve the situation in which, in the absence of errors due to fluctuations in the topology, only the “retrieval” (i.e., identification) of a certain location is taken into account. This upper maximum does not differ from the previous one in terms of the number of different locations erroneously decoded as the correct one, but it modifies the way in which individual decoded locations are distributed around them. Of course all the conditions between these two extremes can be obtained, using an intermediate value of the standard deviation.

We can then define a spatial descriptor of the metric content applicable to spatial representations, as

(15) $$\chi =1-\frac{\sigma }{w}$$

Note that χ = 0 implies that the entire width of the Gaussian in the reduced confusion matrix is due to the poor spatial resolution, as decoding has a standard deviation σ = *w*; χ = 1 instead implies that decoding is spatially exact, and the apparent width w emerges entirely from averaging the errors in the full confusion matrix. We can call χ the metric resolution index.

**Figure 5:**
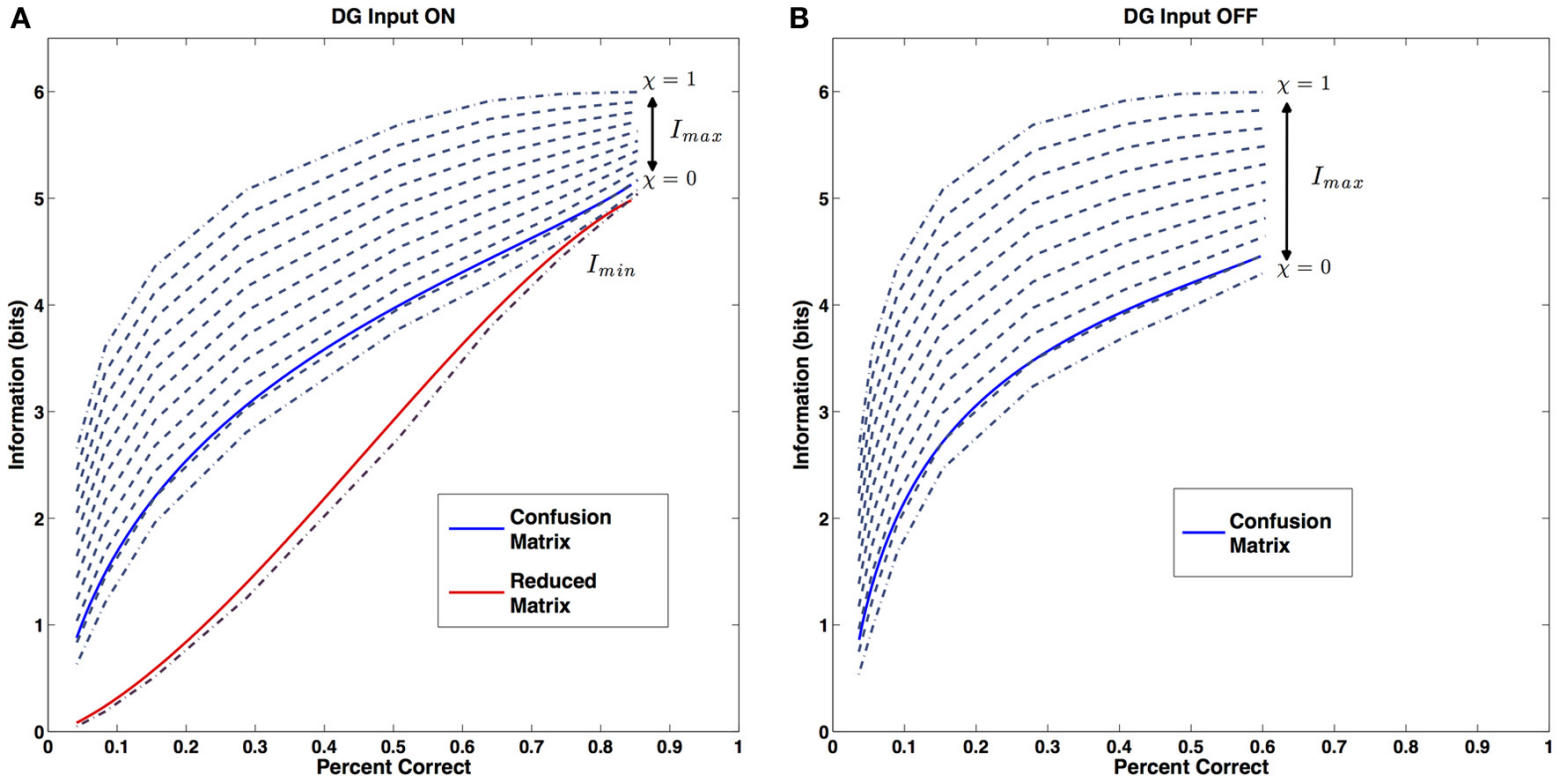
**Metric Resolution. (A)** Metric resolution of the CA3 representation generated with the external DG input. Blue solid line: information content of the confusion matrix (close to χ ~ 0.1). Red solid line: information content of the reduced matrix. Dark red dashed line: theoretical minimum for the information. Dark blue dashed lines: theoretical maxima of the information for progressively decreasing values of the error dispersion. **(B)** Metric resolution of the CA3 representation based on the internal collateral activity only (also close to χ ~ 0.1). Color code, same as **(A)**. Only the metric resolution of the full confusion matrix is shown.

We can see where the data we generated with our simulations sit in relation these three reference curves (Figure 5A). For simplicity and clarity, instead of the original data we will use the fit curves we previously calculated. For each sample size, the parameters of the fit define the two maxima (lowest and highest dark blue dashed curves for χ = 0 and χ = 1, respectively) and the minimum (dark red dashed line) for the information, and we can compare then with the actual value of the simulated network, both the value extracted from the complete confusion matrix (blue solid line), and the value coming from the reduced one (Figure 5A, red solid line). Both series of values appear to sit on a curve of constant metric resolution. The metric resolution expressed by the complete confusion matrix is just above the one defined by our “first maximum” curve, in fact it sits roughly at χ = 0.1. This corresponds to having a matrix in which there are essentially no randomly distributed errors, while those associated with specific locations are spread out locally, with resolution σ = 0.9 w.

As expected, the form of the reduced matrix is quite different. It belongs to a complete different category: here the errors are almost entirely randomly distributed, with the exception of those giving rise to the central bump.

This measure of metric resolution, thus, extends the previous, non-spatial notion of metric content, and captures the qualitatively different nature of the information expressed by the two different types of matrices, full and reduced. It allows us to shed light on the way spatial information is encoded in a CA3-like network by quantifying the presence and the relative importance of the different sources of confusion present in the system. Moreover it indicates that the nature of the confusion is an invariant of the spatial code expressed by the network and it does not depend on the size of the sample of cells used for the decoding.

### Drift

One may ask how the procedure previously described applies to the other condition, in which the DG input is removed. As already shown by Cerasti and Treves (2010) the removal of the external drive causes a major drop in the information about the position of the rat retrievable from the system (also shown in the figure). This drop comes with a parallel decrease in the percent correct obtained by the system (Figure 3B). This effect has been extensively described by Cerasti and Treves (in preparation) and has to do with what there has been called “drift.”

The main effect of storing a map in CA3 is to reduce the number of positions stably represented by the system. After initializing the network in a certain configuration, the activity reverberated in the collaterals modifies this initial pattern and drives it to another, stable point of the configuration space. Eventually, when the final population vector is used to decode the rat position, the outcome will not correspond to the real position. Note that this effect is not due to fast noise, which is kept very low or even absent in the simulations, nor to the use of a limited sample of units: it persists even when the whole population is used in the decoding. Moreover such drift is completely deterministic: at every presentation of the same position, the trajectory of the system's “relaxation” is the same. The size of the distance between the real position and the one decoded after drifting varies for different location of the environment. Its average depends on the number of units in the network.

### Metric resolution without DG input

The main effect of drift on our analysis is to introduce a systematic bias in the decoding of some of the positions in the environment. Since we discretize space in bins of side L, every time drift crosses bin boundaries, the decoded position will belong to a neighboring bin. Not all the points in the same bin are decoded as belonging to another bin, however, some of them will be assigned correctly, while those who are not may move in different directions to different neighboring bins.

This phenomenon introduces a series of non-negligible effects in our measure. It undermines one of the fundamental assumptions of the approach, the unbiased nature of the decoding. In many cases, in those locations where the drift is strong enough, the maximum of the decoding matrix will be associated to an incorrect bin. Moreover it invalidates the use of the reduced matrix to extract information about the distribution of errors around the correct location. Being limited in space, the bump obtained in the reduced matrix is a combination of metric errors and of neighboring bins reached by the drift. This makes the parameters of the bump very unreliable and of little use.

It turns out, however, that the effects of drift can be effectively accommodated in the analysis. To estimate the metric content of the representation generated without the contribution of the external input we follow the same procedure used in the “DG On” condition with a single modification. We calculate the minimal and maximal information levels as in the previous case, using the same parameters extracted from the previous fits and therefore still coming from the reduced matrix of the “DG On” case. The only adjustment we introduce is to impose that each of the 1/*a* bumps present in the confusion matrix is transformed to a combination of two adjacent Gaussian bumps, with the same width of the original, but half of the height. In this way the amount *a* of hits associated with the location is preserved but the drift away from (some of) the locations comprised in the bin is taken into account. This solution is an average of the effects present at different locations in the environment, as in some of them no drift is taking place, while in others the phenomenon is present with different strengths and a variable number of directions.

The result proves that this simple procedure captures very well the effects of drift on the properties of the representation and justifies the 2-bump choice. As shown in Figure 5B, the data sits on a curve of constant metric resolution, which happens to coincide with the one expressed in the previous condition.

The calculation of the metric resolution offers insights on the characteristics of this representation. In fact it shows how, factoring out the drift phenomenon, the representation expressed by the recurrent collaterals alone appears to have the very same metric properties as the one obtained with the active contribution of the DG. For each sample size, the distribution of errors around the central location is indeed the same, as are the number of locations erroneously chosen by the decoding algorithm. As in the previous case, no portion of the errors is allocated randomly. This consistency is noteworthy as we applied a method adapted, in the previous study, to a completely different set of data, where both the information and the percent correct values are drastically different. The metric content analysis shows a remarkable persistence of the relationship between these two quantities, even when the external input is removed.

## Discussion

The ability of the hippocampus to operate as a cognitive map and to successfully guide spatial behavior (Morris et al., 1982) lies in the amount of information about the external environment that can be stored and retrieved from its representations (Battaglia and Treves, 1998; Kali and Dayan, 2000). In particular one would expect that the global topography expressed by these representations, the set of relationships existing among the patterns of activation associated with the different points of the environment, should reflect the one which the animal actually experiences (Curto and Itskov, 2008; Dabaghian et al., 2012). If we imagine an internal observer trying to decode the position of the rat, and the structure of the environment in which it is moving, only relying on the sequence of units that get activated in the hippocampus, similarities between the population vectors would be the most probable criteria to infer the geometrical location of points traversed at different times, or to predict the existence of traversable routes (Gupta et al., 2010; Dragoi and Tonegawa, 2011).

In fact we observe that the topology realized by the averaged activity of our network actually reproduces the torus in which the virtual rat of our simulations is moving. From such a coarse-scale perspective, a CA3 representation is a true reconstruction of the metric the rat is experiencing. Within minor deviations, the correlation between two average population vectors is a good estimate of the distance of the two locations over which their individual population vectors were recorded.

But does such a general approach capture all the variability expressed by the network during its normal operation? We actually find that at a finer scale, the structure of a CA3 spatial representation is richer than it appears at the coarser scale. The difference we observe, between the information content of the full confusion matrix and its reduced version, is telling us about other, non-metric sources of correlation between different parts of the representation. This difference appears when decoding is based on limited samples of network units, but the effects persist up to substantial sizes of the sample, and one should also consider the experimental impracticality of a decoding procedure based on the whole population.

To quantify the properties of the finer aspects of the spatial representation we propose the use of an index, the metric resolution that like the metric content used earlier with non-spatial stimuli, combines the measures of information and percent correct, which are obtained from a confusion matrix. The index allows for an assessment of the metric in the internal representation of space. The measure of metric resolution is applied to our model of a CA3 network in two different regimes, when driven by an external input coming from DG, and when this external input is absent and the active representation is solely an expression of the activity reverberating in the collateral connections. In both cases we find that the metric resolution of the representation is roughly constant, at a value χ ~ 0.1, when the size of the sample is varied, and that the same combination of metric and non-metric structure is present in the confusion matrix generated from these different samples. The metric resolution measure tells us that the self-organizing process has generated an internal representation of space, in the model, which is consistent with external space but has limited metricity, i.e., limited spatial resolution, close to the minimum amount possible (which would yield χ = 0.0).

We acknowledge the possible dependence of the results on the size of the network that represents the real CA3. Although a network of 1500 units is significantly smaller than the actual rodent hippocampus, in some respects it can produce representations which faithfully reproduce the characteristics of the ones experimentally observed (Cerasti and Treves). Moreover the difference in size is less extreme if one regards the real CA3 network as comprised of partially independent sub-modules. The fact that metric content, in our model system, is independent of sample size and of the mode of operation (whether or not externally driven) is an indication of the robustness of this measure.

The errors in decoding that do not depend on the similarity in the representation due to close distance, might be seen as hindering the proper operation of the hippocampal memory system, by introducing ambiguities in the reconstruction of the external space. But another possibility is that these “holes” in the consistency of the representation offer the hippocampus a handle to introduce non-spatial elements of information in the representation itself (Wood et al., 2000; Leutgeb et al., 2005; Singer et al., 2010; Eichenbaum et al., 2012).

Thus the metric content analysis reinforces the suggestion (Cerasti and Treves, submitted) that the CA3 network, with its effectively random drive from the Dentate Gyrus, might be best adapted to serve a memory function, rather than to produce faithful representations of space.

### Conflict of interest statement

The authors declare that the research was conducted in the absence of any commercial or financial relationships that could be construed as a potential conflict of interest.
